# Aberrant static and dynamic functional connectivity of the executive control network in lung cancer patients after chemotherapy: a longitudinal fMRI study

**DOI:** 10.1007/s11682-020-00287-6

**Published:** 2020-04-17

**Authors:** Lanyue Hu, Huiyou Chen, Wen Su, Yujie Zhang, Jia You, Wei Gu, Zhenyu Xiong, Xindao Yin, Yu-Chen Chen

**Affiliations:** 1 Department of Radiology, Nanjing First Hospital, Nanjing Medical University, No.68, Changle Road, Nanjing, 210006 China; 2 Department of Respiratory Medicine, Nanjing First Hospital, Nanjing Medical University, Nanjing, China; 3 Department of Radiation Oncology, The University of Texas Southwestern Medical Center, Dallas, TX USA

**Keywords:** Lung cancer, Static connectivity, Dynamic connectivity, Executive control network, Resting-state fMRI

## Abstract

**Objective:**

The purpose of the current study was to investigate chemotherapy-related variations in the intrinsic static and dynamic functional connectivity (sFC and dFC, respectively) of the executive control network (ECN) in lung cancer patients.

**Materials and methods:**

In this study, we evaluated 18 lung cancer patients scanned before and after adjuvant chemotherapy treatment and compared the patients with 21 healthy controls (HCs). All subjects underwent resting-state functional MRI (rs-fMRI). We constructed the sFC and dFC of the bilateral dorsolateral prefrontal cortex (DLPFC) using a sliding-window approach, and the correlations between the changed sFC or dFC and cognitive performance were analyzed.

**Results:**

Whole-brain sFC analysis showed that the lung cancer patients showed significant FC pattern changes in the bilateral DLPFC, mainly in the bilateral superior frontal gyrus (SFG), bilateral middle frontal gyrus, left superior temporal gyrus, left inferior parietal lobe and the right insula. Furthermore, after chemotherapy, the lung cancer patients showed significantly reduced dFC variability between the right DLPFC and right precuneus compared with HCs. In addition, the decreased dFC between the right DLPFC and left SFG in the lung cancer patients after chemotherapy in state 1 and between the right DLPFC and left insula in the lung cancer patients before chemotherapy in state 2 were negatively correlated with MoCA scores ((r = -0.520, *p* = 0.039; r = -0.548, *p* = 0.028, respectively).

**Conclusions:**

Our results reveal that dynamic connectivity analysis is more effective and sensitive than methods that assume static brain states for linking brain FC patterns and chemotherapy.

## Introduction

Accompanying the increasing morbidity (ranking first among globally diagnosed cancers (Bray et al. 2018)) and overall survival rate of lung cancer, chemotherapy-related cognitive impairment (CRCI) in lung cancer has become a well-recognized side effect during and after adjuvant chemotherapy treatment (Janelsins et al. 2018). Platinum-based chemotherapy was a broadly used global standard of care for advanced lung cancer(Ettinger et al. 2017; Scagliotti et al. 2008). Kaasa et al. (1988) indicated that non-small cell lung cancer patients exhibited cognitive deficits as soon as one month after adjuvant chemotherapy treatment. Longitudinal neuropsychological assessment research studies (Janelsins et al. 2011) suggest that up to 75% of cancer patients experience multiple-domain CRCI, particularly impairments in executive functions, attention, memory and processing speed, during treatment and up to 35% experience persistent CRCI for months or years following treatment completion (Grosshans et al. 2008; Janelsins et al. 2014; Li and Caeyenberghs 2018; Mandelblatt et al. 2013; Pergolizzi et al. 2019). As a major component of advanced cognitive functions, executive functions are vital to human autonomy and are a major determinant of problem behaviors and disabilities (Royall et al. 2002) that incorporate and command more basic cognitive processes (Baddeley 2000; Funahashi and Andreau 2013; Jodzio and Biechowska 2010). Improving our understanding of the changes that occur in executive function-related neurological mechanisms in lung cancer patients receiving adjuvant chemotherapy might be conducive to adjusting treatment regimens appropriately and achieving better functional improvements.

Remarkably, resting-state functional MRI (rs-fMRI) has been proved as a powerful noninvasive method for examining the brain functional network. rs-fMRI evaluates the temporal correlation of intrinsic low-frequency fluctuations based on blood oxygenation level-dependent (BOLD) signals between brain regions during rest(Rosazza and Minati 2011). In practice, using this technique, functional connectivity studies have reported a number of resting-state networks (RSNs) that consist of anatomically separated, but functionally connected regions displaying a high level of correlated BOLD signal activity(van den Heuvel et al. 2008; Van Dijk et al. 2010), and the corroborated executive control network (ECN) mainly involves the dorsolateral prefrontal cortex (DLPFC), dorsal medial prefrontal cortex (DMPFC) and posterior parietal cortex (PPC) (Bressler and Menon 2010; Shirer et al. 2012; Weiland et al. 2014). Recently, a neuroimaging community report showing the altered FC of the ECN in cancer patients during and after chemotherapy has gained increasing attention. Piccirillo et al. (Piccirillo et al. 2015) focused on the FC changes in the frontoparietal network and the cingulo-opercular executive control network in breast cancer patients using a case-control design. Their findings indicated that the standard therapeutic levels of chemotherapy in breast cancer resulted in disrupted FC in the brain networks supporting attention and executive control function. Furthermore, Shelli and colleagues (Kesler et al. 2011) examined differences in prefrontal regions between breast cancer patients received chemotherapy or not and healthy controls. The findings demonstrated hypoactivation in the left medial dorsolateral prefrontal lobe and premotor cortex in breast cancer patients received chemotherapy compared with the healthy controls.

Nevertheless, the aforementioned studies on the FC patterns of the ECN in cancer patients after chemotherapy assumed that FC was stationary during the process of scanning, which is referred to as static FC (sFC). Studies have displayed that the resting brain is an extremely dynamic system and that the functional connectivity states of human brain vary over time (Allen et al. 2014; Hutchison et al. 2013). Thus, the sFC approach has distinct limitations in reflecting dynamic brain processes. Dynamic functional connectivity (dFC) analysis overcomes this static restriction and can reflect the time-varying patterns and temporal dynamic characteristics of FC (Hutchison et al. 2013). Studies have shown that dFC analysis produces more sensitive results than sFC analysis for identifying group differences between healthy controls and patients with various neurological diseases (Chen et al. 2018; Fu et al. 2018; Yang et al. 2019). Notably, a previous study (Kesler et al. 2017) demonstrated that functional dynamics were distinctly lower in breast cancer patients than in control participants.

Previous studies on CRCI in lung cancer have mainly concentrated on brain structure changes and default mode network disruption (Bromis et al. 2017; Simo et al. 2015, 2016, 2018; Welzel et al. 2008). Bromis et al. (2017) found significant FC disruptions within all the rest-state networks of lung cancer patients after chemotherapy compared with the healthy controls. Additionally, other results suggest that the reduced rest-state FC pattern within the default mode network (DMN) and the cognitive disorder were associated with cancer and chemotherapy in lung cancer patients (Simo et al. 2018; Zhang et al. 2019). Unfortunately, executive function disorders related to cancer and chemotherapy in the lung cancer patient population remain poorly understood, and further explorations are urgently necessary.

The present study aimed to investigate cancer- and chemotherapy-related intrinsic sFC and dFC differences within the ECN in rs-fMRI employing the seed-based approach in lung cancer survivors. The prefrontal cortex has been demonstrated to be an important structure for executive functions(Funahashi and Andreau 2013). Moreover, the DLPFC, in the middle frontal gyrus, which is the core node of the ECN, is involved in functions including working memory, prospective memory and executive function (Mitchell et al. 2008; Pochon et al. 2001). Based on previous neuroimaging findings, we selected the bilateral DLPFC as the critical seed region and hypothesized that (1) sFC and dFC patterns in the DLPFC are significantly functionally disrupted in lung cancer patients after chemotherapy and (2) that altered sFC or dFC was associated with cognitive performance in lung cancer patients. As far as we know, this is the first longitudinal study to explore links between sFC and dFC changes in ECN patterns and chemotherapy in the lung cancer patient population.

## Materials and methods

### Participants

The prospective study was approved by the Research Ethics Committee of Nanjing Medical University, and all subjects signed informed consent before their participation in the study protocol.

This was a longitudinal study of lung cancer patients scheduled to incorporate scans at baseline (t0) and three to six months (t1) after receiving adjuvant chemotherapy and to include age/sex-matched healthy controls (HCs). Eighteen lung cancer patients from the Department of Respiratory Medicine, Nanjing First Hospital and 21 healthy controls (aged between 48 and 70 years, received at least 6 years of education) were enrolled through online advertisements and age, sex, and education level-matched between May 2018 and August 2019. All participants were right-handed. Among the patients treated with chemotherapy (platinum-based doublet with pemetrexed), 10 patients received cisplatin-based therapy, and 8 patients received carboplatin-based therapy for three to six months. No participants were excluded from the fMRI analysis because of excessive head motion during scanning. The exclusion criteria for all participants were: receipt prophylactic cranial irradiation; presence of a metastatic brain tumor; declared history of known stroke, craniocerebral trauma, epilepsy, Alzheimer’s disease, Parkinson’s disease, other acute psychiatric or neurological illnesses; presence of a major medical illness (e.g., anemia, severe heart diseases, thyroid dysfunction or abnormality in liver or kidney function); and presence of severe vision or hearing loss. The neuropsychological status and general cognitive function of the participants were established using the Mini Mental State Exam (MMSE) (Galea and Woodward 2005) and Montreal Cognitive Assessment (MoCA) (Nasreddine et al. 2005).

### MRI acquisition

All MRI data were acquired in 3.0 T MRI scanner (Ingenia, Philips Medical Systems, Netherlands) with an 8-channel receiver array head coil and parallel imaging was employed. Tight but comfortable foam padding was used to minimize head motion, and earplugs were used to alleviate scanner noise. Subjects were instructed to lie with their eyes closed and stay awake, to not thinking things in particular. For each participant, routine MRI sequences, including thick-slice T2- and T1-weighted imaging as well as T2 fluid-attenuated inversion recovery (FLAIR) imaging, were performed to ensure that there were no visible brain lesions or brain metastases.

The resting state fMRI data were obtained with the gradient echo-planar imaging sequence as follows: repetition time (TR) = 2000 ms; echo time (TE) = 30 ms; slices = 36; thickness = 4 mm; gap = 0 mm; field of view (FOV) = 240 mm × 240 mm; acquisition matrix = 64 × 64; and flip angle (FA) = 90°. High-resolution three-dimensional T1-weighted images (3D-T1WI) data were acquired with magnetization-prepared rapid gradient-echo sequence as follows: TR = 8.1 ms; TE = 3.7 ms; slices = 170; thickness = 1 mm; gap = 0 mm; FA = 8°; acquisition matrix = 256 × 256; and FOV = 256 mm × 256 mm. The resting state fMRI sequence lasted 488 s, and the structural sequence lasted 329 s.

### Data preprocessing

Functional preprocessing was performed using Statistical Parametric Mapping (SPM8; http://www.fil.ion.ucl.ac.uk/spm) and the Graph Theoretical Network Analysis Toolbox for Imaging Connectomics (GRETNA) (Wang et al. 2015) (2.0.0A http://www.nitrc.org/projects/gretna/). The processing pipeline included the following stages: (i) The first 10 volumes were discarded to calculate the time required for participants to adapt to the scanning environment. (ii) Slice timing corrected and realigned were performed for the remaining 220 images, and head motion > 2.0-mm in each direction or rotation angle > 2.0° were removed from analysis. (iii) The remaining dataset was normalized to the Montreal Neurological Institute (MNI) EPI template (reslicing voxel size as 3 × 3 mm^3^). (iv) Spatially smoothing with a Gaussian kernel [6 mm full width at half maximum (FWHM)]. (v) Detrending and filtering (0.01–0.08 Hz) were performed in turn. Subsequently, several nuisance signals including head motion, the global mean, and signals from white matter (WM) and the cerebrospinal fluid (CSF) were regressed from the data.

### Static FC analysis

The seed regions in the bilateral DLPFC were extracted from the Brodmann template using WFU Pick Atlas software (Maldjian et al. 2003). For sFC analysis, the mean time series of the signal in the bilateral DLPFC were obtained to serve as the seed time course, and the Pearson correlation coefficients (r) were finally calculated between the mean time series of each seed region and all brain voxel time series. Finally, a Fisher’s z-transform was used to obtain variables approximating a normal distribution (Lowe et al. 1998).

### Dynamic FC analysis

Dynamic FC analysis was performed using the Dynamic Brain Connectome (Dynamic BC) toolbox (Liao et al. 2014) (V2.1 http://restfmri.net/forum/DynamicBC). Temporal dynamic patterns were characterized by using a sliding-window approach, which was created by convolving a rectangle with a Gaussian kernel (σ = 3TRs). Previous studies have revealed that a frequency interval of [0 − 1/w] Hz should be the target due to the low-pass filtering effect of the window and that the minimum window length should be above 1/f_min_ (Leonardi and Van De Ville 2015). Some researchers have applied a sliding-window length of as small as 10 s (Thompson et al. 2013) and as long as 180 s (Gonzalez-Castillo et al. 2015). Thus, the window size was chosen to be 20 TRs (40 s), and the window overlap was selected to be 95% (window-shifting step size of 1 TR), resulting in W = 201 windows (Chen et al. 2017). The temporal correlation coefficient between the time courses of each seed and that for the other brain voxels was calculated for each sliding window. For each subject, a range of sliding-window correlation maps was acquired. To characterize the time variability in FC, the standard deviation of each voxel across a number of windows was calculated and the Fisher Z-transformed used to acquire variables similar to a normal distribution (Liao et al. 2018).

### Clustering analysis

To assess reoccurring dFC patterns, a k-means clustering algorithm was used to analyze dFC estimates of all subjects (combining the lung cancer and HC groups) (Allen et al. 2014). We used the L1 distance function to assess the similarity between sliding window FCs, as L1 distance has been proved to be an effective measurement method for high-dimensional data (Aggarwal et al. 2001; Allen et al. 2014). We determined the number of clusters to be three using the elbow criterion of the cluster validity index, which is computed as the ratio between the within-cluster distance and between-cluster distance. The gained clustering centroids were then served as the departure points to cluster all dFC windows from all subjects.

### Statistical analysis

SPSS software (version 19.0; SPSS, Chicago, IL) was used for statistical analyses, and a corrected statistically significance level of p < 0.05 was obtained. Group comparisons of demographic information or clinical measures were performed using two-sample t-tests to compare continuous data between the lung cancer groups and HC group.

A one-sample t-test was performed to analyze individual sFC maps in a voxel wise manner. The test was used to determine the sFC patterns in regions with significant connectivity to the specific seeds in each group. Two-sample t-tests were then performed to determine the sFC and dFC differences between HCs and lung cancer patients at t0 and t1, and paired t-tests were used to estimate the sFC and dFC differences between t0 and t1 in the lung cancer patients. Correction was implemented using false discovery rate (FDR) approach and significance threshold was set at p < 0.01. Age, sex and education were used as nuisance covariates to control for the effects of these factors on the results.

In order to study the temporal properties of dFC patterns, we calculated some dynamic indicators including the mean dwell time (MDT) and the number of transitions (NT). The average time (in windows) spent in a state before transitioning to another state was defined as the MDT. In addition, the NT stands for the number of transitions between states. Group differences tests in the MDT and NT between HCs and lung cancer patients at t0 and t1were checked using a two-sample t-test (P < 0.01, FDR correction). Between-group differences among the lung cancer patients at t0 and t1 were investigated using paired t-tests (P < 0.01, FDR correction).

To evaluate the correlational relationship of abnormal sFC or dFC and cognitive performance, Pearson correlation analyses were implemented by using SPSS19.0. P less than 0.05 was considered statistically significant. Partial correlations were calculated after correcting for age, sex and education.

## Results

### Demographic and neuropsychological data

All the detailed demographics and a summary of the histological diagnosis and tumor stage are included in Table 1. There was no significant difference in age, sex, education level, or MMSE score between the lung cancer patients and the HCs (all p > 0.05). The lung cancer patient group at t1 had worse MoCA scores than the HC group and lung cancer patient group at t0 (p < 0.001).

**Table 1:** Demographic and clinical characteristics of all subjects

	Lung cancer (n = 18)		HCs (n = 21)	*p* value
Age, year	59.72 ± 9.04		58.57 ± 9.61	0.703^a^
Education, year	10.59 ± 4.25		10.71 ± 1.88	0.904^a^
Gender, male/female	13/5		13/8	0.828^b^
Histological diagnosis				
Adenocarcinoma	9(50)			
Squamous cell carcinoma	7 (39)			
SCLC	2 (11)			
Tumor stage				
Limited disease	1 (5)			
Extensive disease	1 (5)			
I	3 (17)			
II	5(28)			
III	4 (22)			
IV	4 (22)			
	t0	t1		
MMSE	26.78 ± 1.17	26.61 ± 1.19	27.19 ± 1.03	0.248^a1^;0.163^a2^;0.698^a3^
MoCA	22.22 ± 1.93	22.06 ± 2.94	25.76 ± 1.61	0.000^*a1^; 0.000^*a2^;0.817^a3^

Data are expressed as Mean±SD, n (%) or median (range), ^a^The P values are obtained by using two sample t-test; ^b^The P values are obtained by using χ2 test. ^*^P<0.05 is considered significant. ^a1^ The P value between lung cancer patients before chemotherapy and HCs;^a2^ The P value between lung cancer patients after chemotherapy and HCs;^a3^ The P value between lung cancer patients before and after chemotherapy. MMSE, Mini Mental State Exam; MoCA, Montreal Cognitive Assessment; HCs, healthy controls

### Static FC results

#### Within-group comparisons

**Fig. 1:**
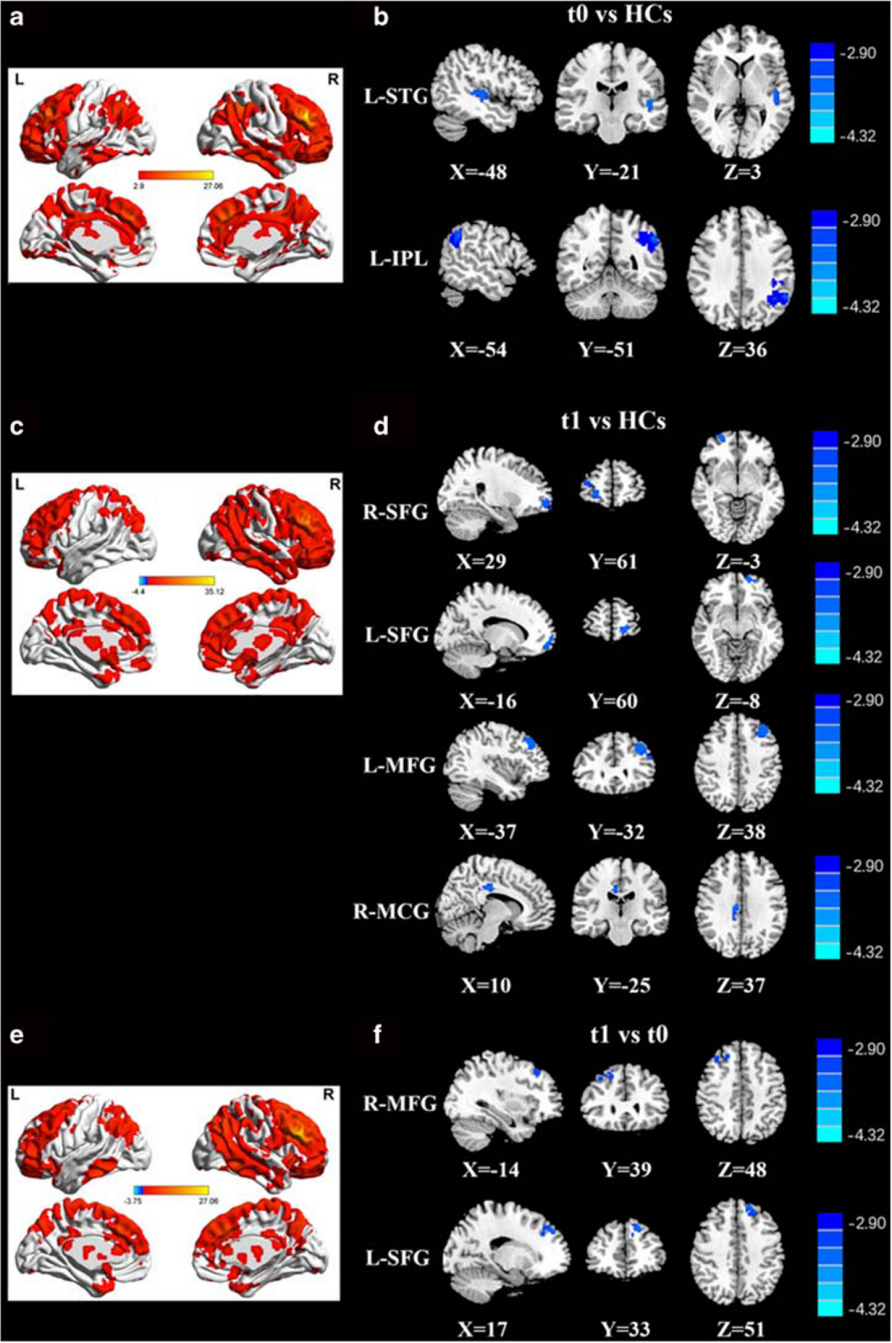
**A**, **C**, **E** Visualization of significant FC patterns of the right DLPFC by whole-brain scanning using a one-sample t-test to compare HCs and lung cancer patients at t0 and t1. **B** Compared with HCs, lung cancer patients at t0 showed decreased FC between the R-DLPFC and left STG or left IPL. **D** Compared with the HCs, the lung cancer patients at t1 exhibited reduced FC between the R-DLPFC and bilateral SFG, left MFG, or right MCG. **F** Compared with those at t0, the lung cancer patients at t1 showed reduced FC between the R-DLPFC and right MFG or left SFG. Significance thresholds were corrected using an FDR criterion and set at p < 0.01. HCs: healthy controls; t0: lung cancer patients before chemotherapy; t1: lung cancer patients after chemotherapy

Individual sFC maps for each group are shown in Fig. 1. The bilateral DLPFC mainly displayed positive FC within the ECN regions, mainly involving the prefrontal cortex (PFC), PPC, the temporal cortex, and some subcortical regions, in HCs (Fig. 1A) and lung cancer patients at t0 and t1 (Fig. 1C and E).

#### Between-group comparisons

**Fig. 2:**
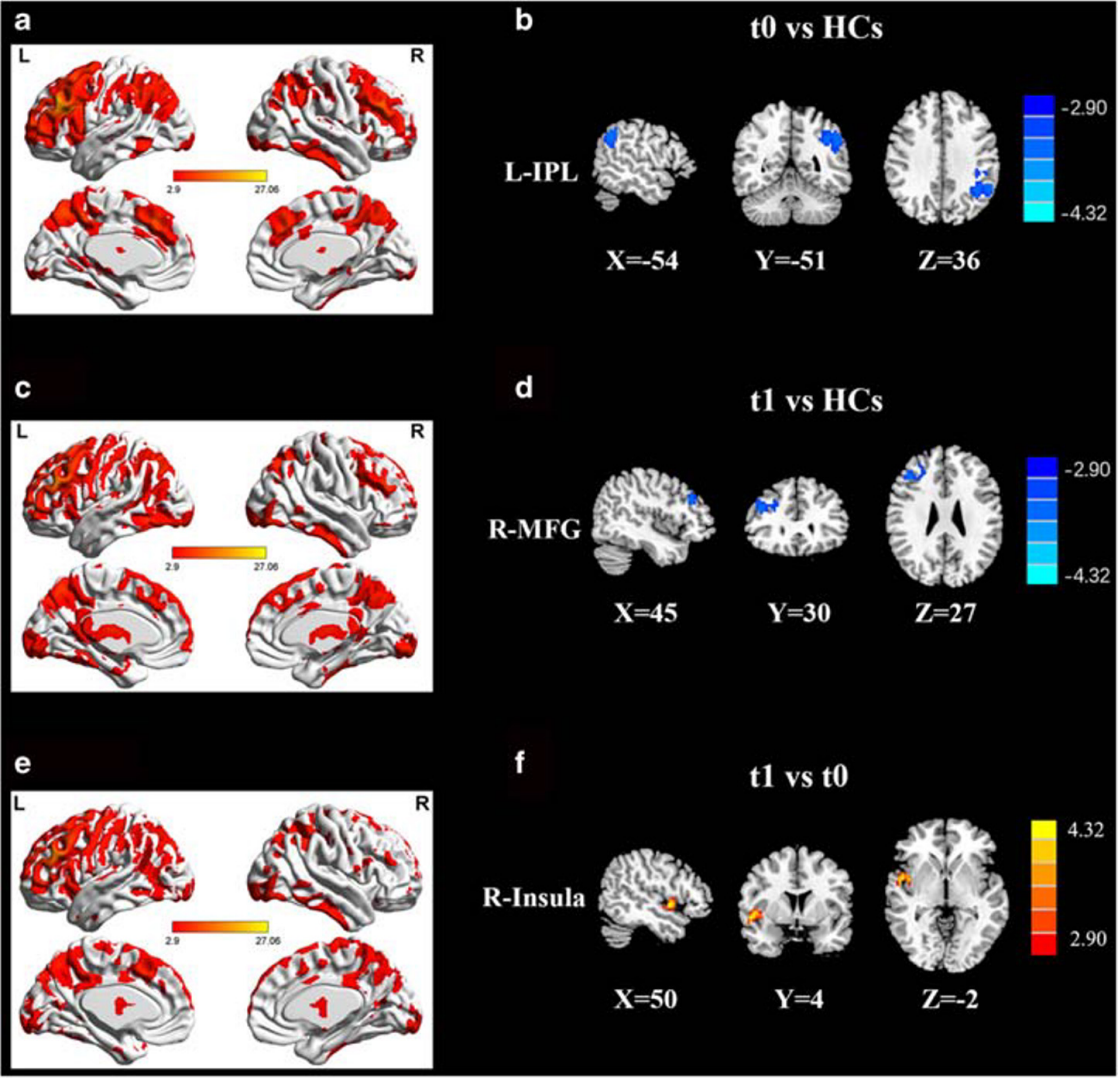
**A**, **C**, **E** Significant FC patterns in the left DLPFC determined by whole-brain scanning using a one-sample t-test to compare healthy controls and lung cancer patients at t0 or t1. **B** Compared with the HCs, the lung cancer patients at t0 showed decreased FC between the L-DLPFC and left IPL. **D** Compared with the healthy controls, the lung cancer patients at t1 exhibited reduced FC between the L-DLPFC and right MFG. **F** Compared with those at t0, the lung cancer patients at t1 showed increased FC between the L-DLPFC and right insula. Significance thresholds were corrected using an FDR criterion and set at p < 0.01. HCs: healthy controls; t0: lung cancer patients before chemotherapy; t1: lung cancer patients after chemotherapy

Seed-based whole-brain sFC analysis revealed the sFC patterns of lung cancer patients and HCs (Table 2; Figs. 1 and 2). Based on the seeds of the bilateral DLPFC, relative to the HCs, the lung cancer patients at t0 revealed marked decreased sFC between the right DLPFC and left inferior parietal lobule (IPL) or left superior temporal gyrus (STG) and between the left DLPFC and left IPL; the lung cancer patients at t1 showed significant reductions in sFC between the right DLPFC and bilateral SFG, left middle frontal gyrus (MFG), or right medial cingulate gyrus (MCG) and between the left DLPFC and right MFG. Compared with those at t0, the lung cancer patients at t1 showed decreased sFC between the right DLPFC and left SFG and increased sFC between the left DLPFC and right insula.

**Table 2:** Abnormal static functional connectivity between groups

ROIs	Group comparisons	Brain region	BA	MNI Coordinates x, y, z (mm)	T score	Voxels
R-DLPFC	t0 vs. HCs	Temporal_Sup_L	81	-48, -21, 3	-4.2286	89
		Parietal_Inf_L	61	-54, -51,36	-5.8758	315
	t1 vs. HCs	Frontal_Sup_R	6	24, 57, -6	-4.4452	109
		Frontal_Sup_L	5	-15, 60, -9	-4.1401	52
		Frontal_Mid_L	7	-39,30,42	-4.689	145
		Cingulum_Mid_R	34	9, -21,33	-4.1414	65
	t1 vs. t0	Frontal_Sup_L	3	-18, 45, 22	-4.5187	85
		Frontal_Mid_R	8	45, 33, 51	-3.7625	45
L-DLPFC	t0 vs. HCs	Parietal_Inf_L	61	-54, -51,36	-4.907	315
	t1 vs. HCs	Frontal_Mid_R	8	45,30,27	-3.8228	176
	t1 vs. t0	Insula_R	30	51,3, -3	4.6003	84

Thresholds were set at a corrected p < 0.01 corrected by FDR criterion. BA, Brodmann’s area; MNI, Montreal Neurological Institute; L, left; R, right; t0, lung cancer patients before chemotherapy; t1, lung cancer patients after chemotherapy; HCs, healthy controls

### Dynamic FC results

#### Dynamic FC variability

**Fig. 3:**
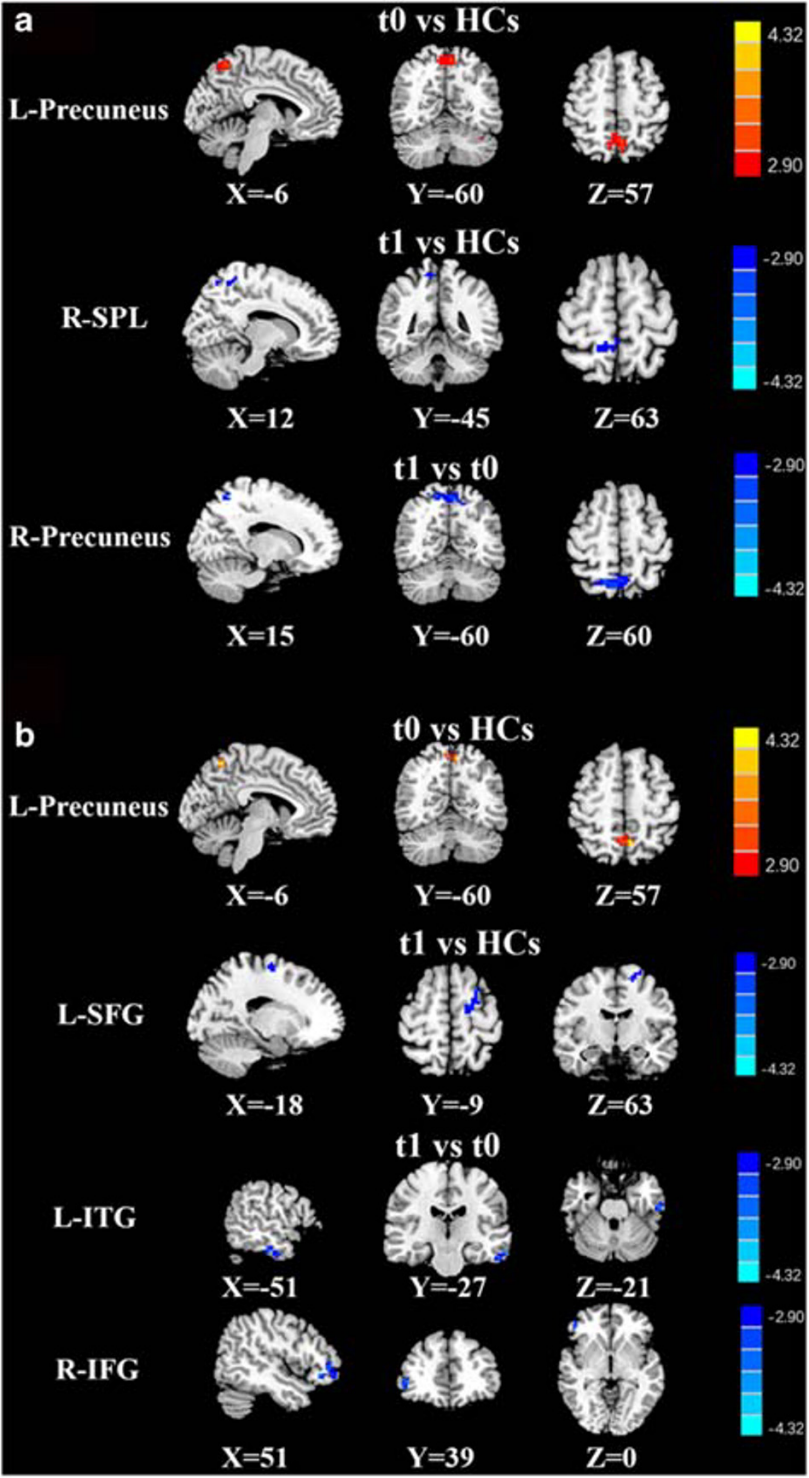
Brain regions with significant intergroup differences in dFC variability. **A** Using the right DLPFC as the seed region, significant group differences were detected in multiple brain regions. **B** Using the left DLPFC as the seed region, significant group differences were detected in multiple brain regions. Significance thresholds were corrected using an FDR criterion and set at p < 0.01

The differences in dFC variability between the lung cancer patient groups and the HC group are illustrated in Fig. 3; Table 3. Relative to the HCs, the lung cancer patients at t0 exhibited significantly increased dFC variability between the bilateral DLPFC and left precuneus; at t1, significantly reduced dFC variability between the right DLPFC and right superior parietal lobule (SPL) and between the left DLPFC and left SFG was revealed. In addition, paired t-tests showed the lung cancer patients at t1 exhibited significantly decreased dFC variability between the right DLPFC and right precuneus, the left DLPFC and right inferior frontal gyrus (IFG) or left inferior temporal gyrus (ITG).

**Table 3:** Abnormal dynamic functional connectivity between groups

ROIs	Group comparisons	Brain region	BA	MNI Coordinates x, y, z (mm)	T score	Voxels
R-DLPFC	t0 vs. HCs	Precuneus_L	67	-6,-60,57	5.0394	77
	t1 vs. HCs	Parietal_Sup_R	60	12,-45,63	-3.6612	43
	t1 vs. t0	Precuneus_L	67	15,-60,60	-5.686	116
L-DLPFC	t0 vs. HCs	Precuneus_L	67	-6,-60,57	5.0394	77
	t1 vs. HCs	Frontal_Sup_L	3	-18 -9,63	-4.2194	42
	t1 vs. t0	Temporal_Inf_L	89	-57,-21,-27	-4.2989	43
		Frontal_Inf_R	16	51,39,0	-4.4814	44

Thresholds were set at a corrected p < 0.01 corrected by FDR criterion. BA, Brodmann’s area; MNI, Montreal Neurological Institute; L, left; R, right; t0, lung cancer patients before chemotherapy; t1, lung cancer patients after chemotherapy; HCs, healthy controls

#### Dynamic connectivity states and the associated brain dynamic FC

**Fig. 4:**
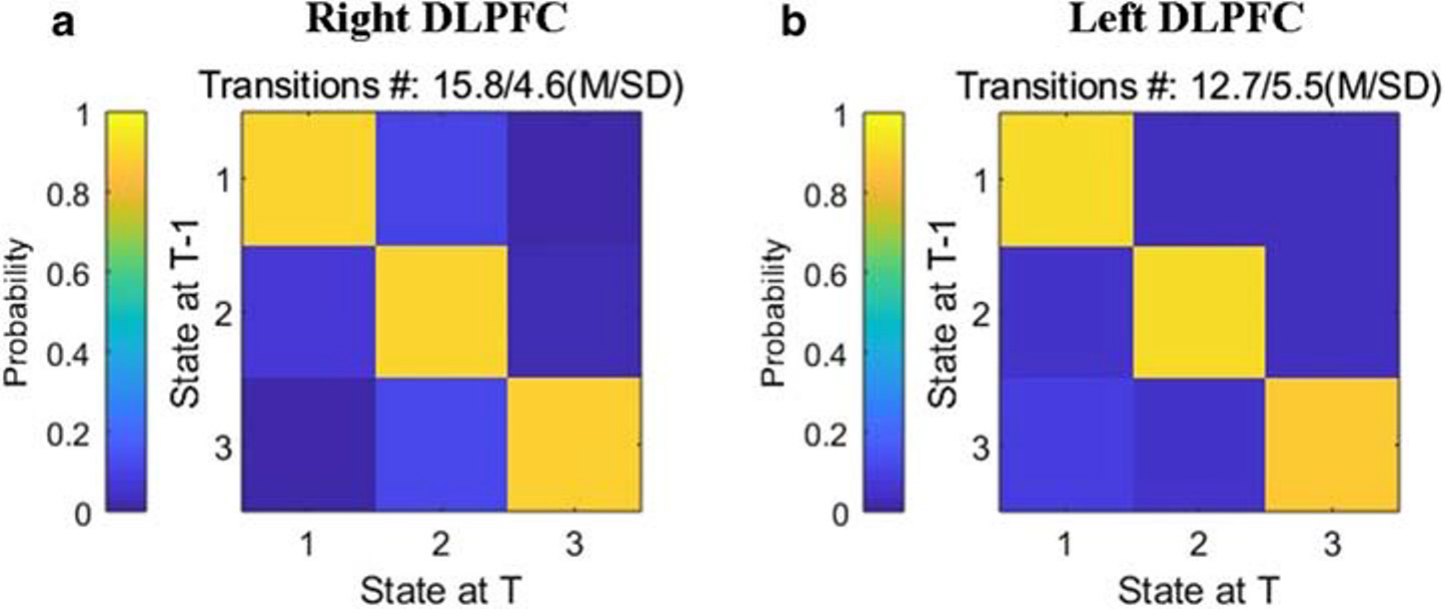
Transition matrices of different states, which were extracted using the K-means clustering method with a cluster size of 3. **A** The transition matrices of different states based on the right DLPFC as the seed region. **B** The transition matrices of different states based on the left DLPFC as the seed region

**Fig. 5:**
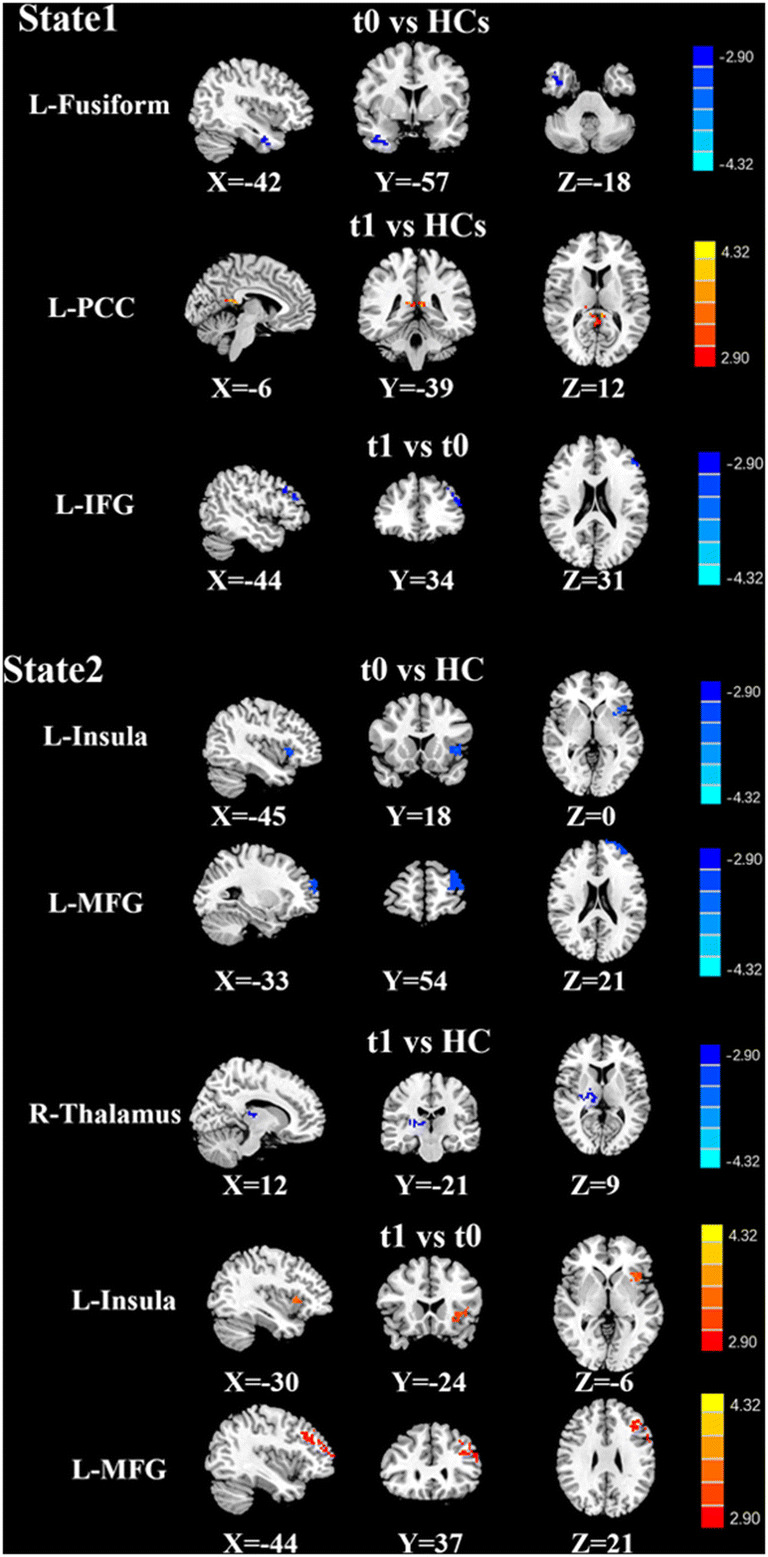
Dynamic FC pattern based on the right DLPFC in each state, where lung cancer patients had a reduced or increased FC pattern in comparison to healthy controls. Significance thresholds were corrected using an FDR criterion and set at p < 0.01

**Fig. 6:**
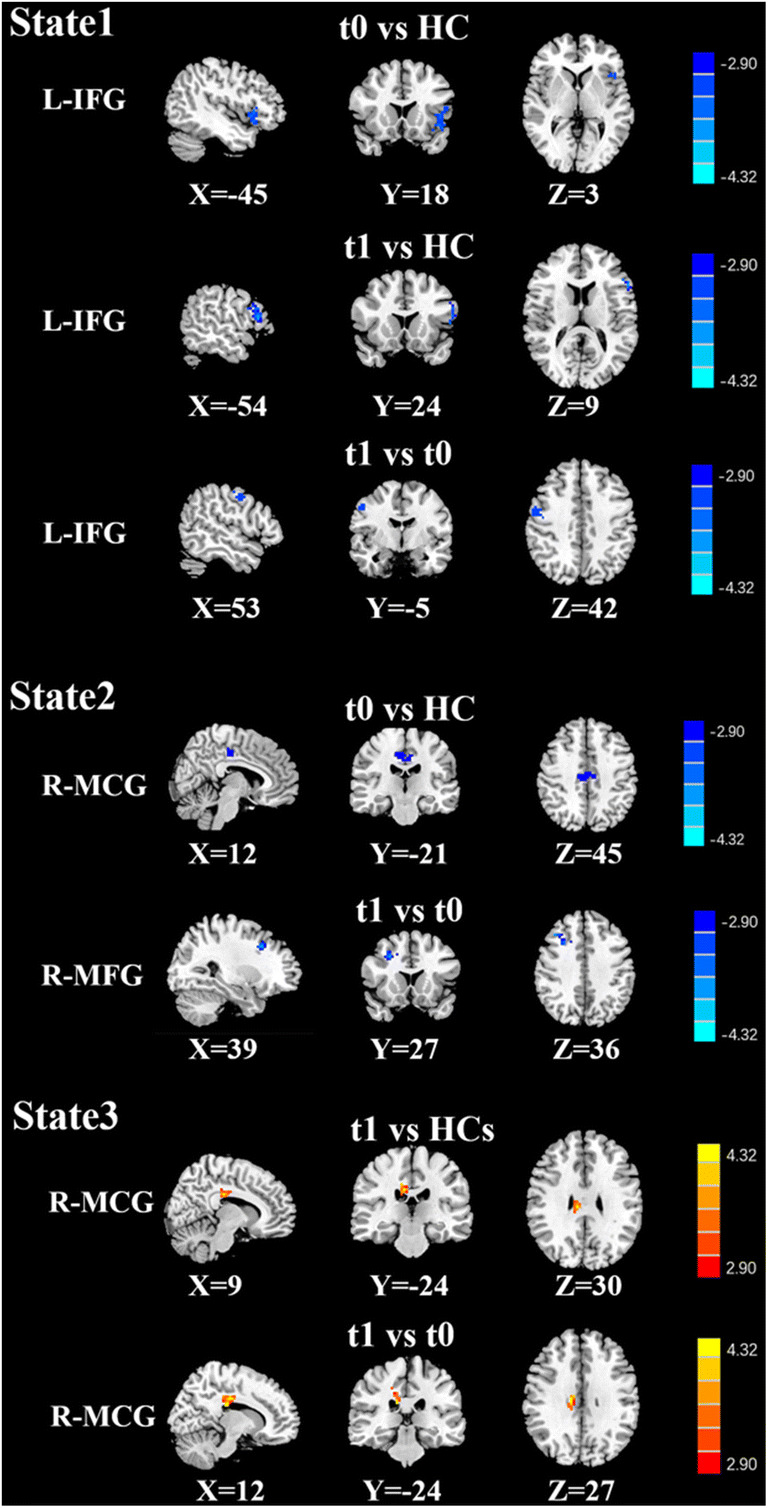
Dynamic FC pattern based on the left DLPFC in each state, where lung cancer patients had a reduced or increased FC pattern in comparison to healthy controls. Significance thresholds were corrected using an FDR criterion and set at p < 0.01

Figure 4 shows the transition matrices of different states, which were extracted using the K-means clustering method with a cluster size of 3. The different FCs between groups in each state are displayed in Figs. 5 and 6; Tables 4 and 5.

**Table 4:** Abnormal dynamic functional connectivity of R-DLPFC at different states between groups

States	Group comparisons	Brain region	BA	MNI Coordinates x, y, z (mm)	T score	Voxels
State 1	t0 vs. HCs	Fusiform_L	55	-42,-57,-18	-2.8578	45
	t1 vs. HCs	Cingulum_Post_L	35	-6,-39,12	3.2144	42
	t1 vs. t0	Frontal_Sup_L	3	-48,36,21	-4.8199	58
State 2	t0 vs. HCs	Insula_L	30	-45,18,0	-3.5169	62
		Frontal_Mid_L	7	-33,54,21	-3.2704	106
	t1 vs. HCs	Thalamus_R	78	12,-21,9	-3.4867	66
	t1 vs. t0	Insula_L Frontal_Mid_L	29 7	-30,24,-6 -42,42,24	3.9534 4.4502	61 193

Thresholds were set at a corrected p < 0.01 corrected by FDR criterion. BA, Brodmann’s area; MNI, Montreal Neurological Institute; L, left; R, right; t0, lung cancer patients before chemotherapy; t1, lung cancer patients after chemotherapy; HCs, healthy controls

**Table 5:** Abnormal dynamic functional connectivity of L-DLPFC at different states between groups

State	Group comparisons	Brain region	BA	MNI Coordinates x, y, z (mm)	T score	Voxels
State 1	t0 vs. HCs	Frontal_Inf _L	15	-45,18,3	-3.5752	85
	t1 vs. HCs	Frontal_Inf _L	13	-54,24,9	-2.6263	50
	t1 vs. t0	Frontal_Sup _R	6	24,69,-3	-3.9716	71
State 2	t0 vs. HCs	Cingulum_Mid_R	34	12,-21,45	-3.6457	54
	t1 vs. t0	Frontal_Mid_R	8	39,27,36	-4.1514	45
State 3	t1 vs. HCs	Cingulum_Mid_R	34	9,24,30	3.5843	56
	t1 vs. t0	Cingulum_Mid_R	34	12,-24,27	3.6361	60

Thresholds were set at a corrected p < 0.01 corrected by FDR criterion. BA, Brodmann’s area; MNI, Montreal Neurological Institute; L, left; R, right; t0, lung cancer patients before chemotherapy; t1, lung cancer patients after chemotherapy; HCs, healthy controls

Based on the seeds of the bilateral DLPFC, in state 1, compared with the HCs, the lung cancer patients at t0 showed significantly decreased dFC between the right DLPFC and the left fusiform and between the left DLPFC and left inferior orbitofrontal gyrus; the lung cancer patients at t1 showed significantly increased dFC between the right DLPFC and the left posterior cingulate cortex (PCC) and decreased dFC between the left DLPFC and triangle inferior frontal gyrus (IFG). Compared with those at t0, the lung cancer patients at t1 exhibited significantly decreased dFC between the right DLPFC and the left IFG and between the left DLPFC and right superior orbit frontal gyrus. In state 2, relative to the HCs, the lung cancer patients at t0 showed significantly decreased dFC between the right DLPFC and left insula or MFG and between the left DLPFC and right MCG; at t1, the lung cancer patients showed significantly reduced dFC between the right DLPFC and left thalamus. Compared with those at t0, the lung cancer patients at t1 showed significantly increased dFC between the right DLPFC and left insula or left MFG and decreased dFC between the left DLPFC and right MFG. In state 3, the lung cancer patients at t1 showed significantly increased dFC between the left DLPFC and right MCG compared with those at t0 and the HCs.

**Fig. 7:**
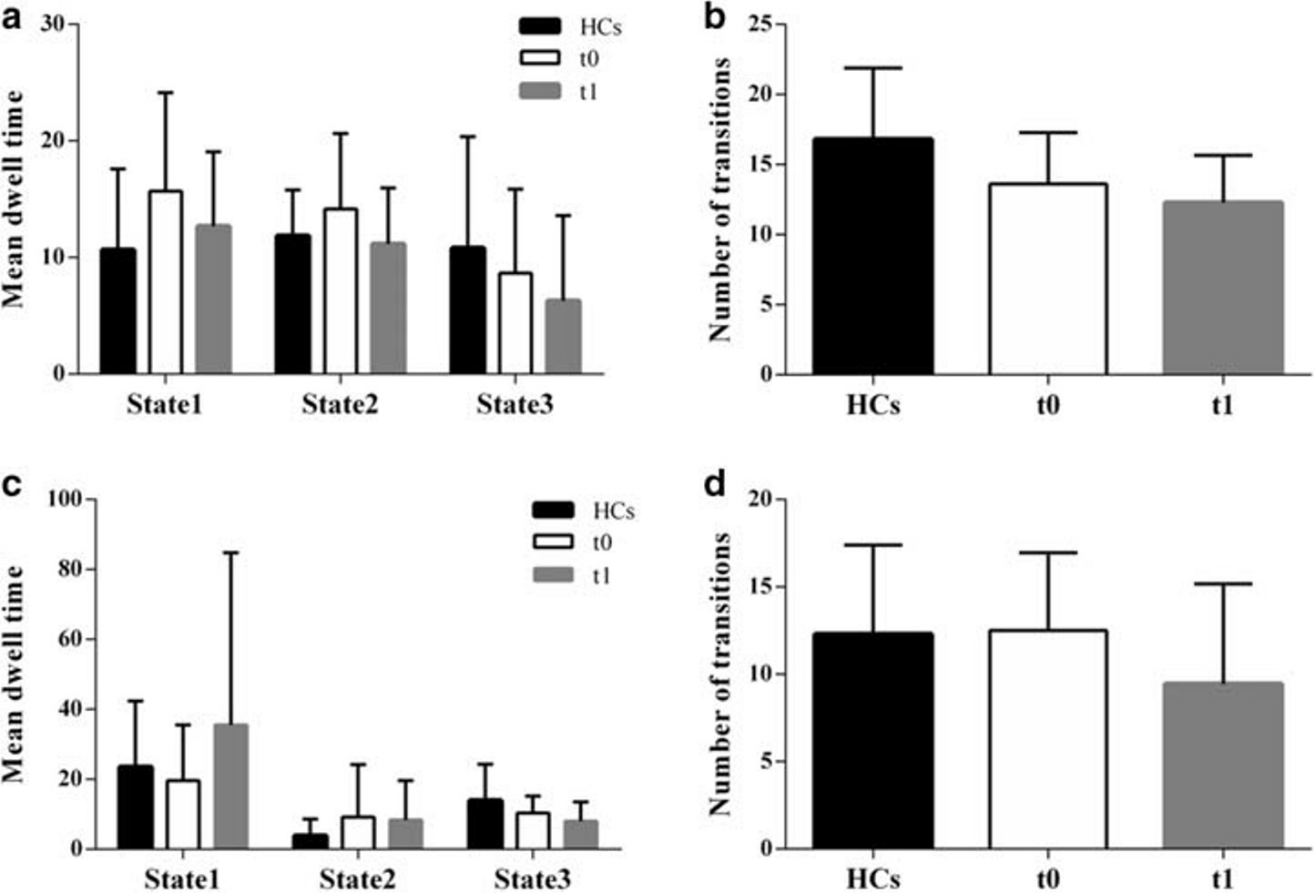
Temporal properties of dFC states in the lung cancer patient and healthy control groups. **A** and **C** Mean dwell time; **B** and **D** number of transitions between states. HCs: healthy controls; t0: lung cancer patients before chemotherapy; t1: lung cancer patients after chemotherapy

Based on the seed of the right DLPFC, in regard to the between-group comparisons of the MDT and NT in each state, we have found that the lung cancer patients at t0 seemed to stay in state 1 for a shorter time (t=-2.045, *p* = 0.048) and that the lung cancer patients at both t0 and t1 appeared to have a lower NT (t=-2.274, *p* = 0.029; t=-3.329, *p* = 0.003) than the HCs (Fig. 7A, B).

Based on the seed of the left DLPFC, for the between-group comparisons of the MDT and NT in each state, we found that the lung cancer patients after chemotherapy seemed to stay in state 3 for a shorter time than the HCs(t=-2.292, *p* = 00,029), and paired t-tests showed that the lung cancer patients at t1 had a lower NT than those at t0 (t=-2.233, *p* = 0.039) (Fig. 7C, D).

### Correlation analysis results

**Fig. 8:**
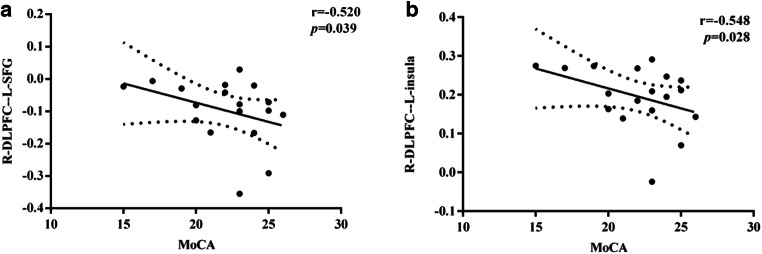
**A** Negative correlation between the decreased dFC between the right DLPFC and left SFG and MoCA scores in patients after chemotherapy compared with nonchemotherapy patients (r = -0.520, p = 0.039). **B** Negative correlation between the reduced dFC between the right DLPFC and left insula and MoCA scores in prechemotherapy patients compared with healthy controls (r = -0.548, p = 0.028)

There were no significant correlational relationships between sFC and MoCA scores at t0 or t1 or in the HCs. In state 1, in comparisons within the lung cancer groups at t1 versus t0, the decreased dFC of the right DLPFC to the left SFG was negatively correlated with MoCA scores (r=-0.520, *p* = 0.039) (Fig. 8A). Moreover, in state 2, compared with that in the HCs, the decreased dFC of the right DLPFC to the left insula in the lung cancer group at t0 was negatively associated with reduced MoCA scores (r=-0.548, *p* = 0.028) (Fig. 8B).

## Discussion

CRCI has been quantified using neuropsychological test batteries, and medical imaging research using rs-fMRI has attempted to elucidate the underlying neurobiological mechanisms. Indeed, research on rest-state sFC changes related to chemotherapy has witnessed exponential growth. In the current study, we found that the regions interacting with the bilateral DLPFC were mainly distributed in the PFC, partial parietal and temporal lobe. The DLPFC, which is the core node of the ECN, is involved in functions including working memory, prospective memory and executive function (Barbey et al. 2013; Pochon et al. 2001). The PFC and its cortical and subcortical connections play a central role in processing executive functions (Rusnakova et al. 2011). In addition, the STG is considered to be a central brain region involved in processing executive functions together with frontal areas that form a cognitive network (Bockova et al. 2007). Recent studies have demonstrated that the PFC and STG are impaired in lung cancer patients after chemotherapy (McDonald et al. 2012; Simo et al. 2018; Wang et al. 2016). In breast cancer patients, reduced FC was chiefly seen between the right DLPFC and the right IFG, right MFG, and left STG compared with HCs (Wang et al. ). One month after standard chemotherapy, McDonald et al. (McDonald et al. ) reported that both patients receiving chemotherapy and those not requiring chemotherapy showed decreased activation of the IFG compared to HCs. Our findings are not only consistent with literature reports but are also unique. Our findings support the idea that the cancer disease process itself affects brain functioning (Menning et al. 2017); in addition, by means of longitudinal comparison, we confirmed the exact effect of chemotherapy on sFC.

Furthermore, the decreased sFC between the bilateral DLPFC and the left IPL was demonstrated in prechemotherapy lung cancer patients compared with HCs. Sanne et al. (Menning et al. 2017) detected that parietal activation decreased in a breast cancer group that was not exposed to any systemic treatment compared to an HC group. Based on previous studies, we speculated that the parietal areas are more susceptible to factors related to stressful events, such as cancer diagnosis and surgery. In contrast, the lung cancer group showed significantly increased sFC between the left DLPFC and right insula after chemotherapy. The insula is believed to be involved in emotion and consciousness. A previous study (Feng et al. 2019) reported that patients treated with chemotherapy showed persistent anxiety and depression symptoms over time and that psychological symptom score changes were significantly positively associated with FC changes between the left hippocampus and left insula. We hypothesized that the increased sFC between the left DLPFC and right insula may be a mechanism of self-regulating negative emotion in lung cancer patients.

Previous studies have demonstrated altered brain sFC patterns in the ECN induced by cancer and chemotherapy. Recent evidence has shown that exploiting the dynamic properties of FC instead of traditional sFC metrics can open up novel thinking for interpreting brain functioning on different timescales (Liegeois et al. 2019). Shelli R. Kesler and his colleagues (Kesler et al. 2017) demonstrated that functional dynamics were significantly lower in patients with breast cancer than in control participants. In this study, dFC provided additional information that was different from but complementary to the information for sFC. We found significantly decreased dFC variability between the bilateral DLPFC and right SPL and left SFG in lung cancer patients after chemotherapy and significantly reduced dFC between the bilateral DLPFC and left precuneus and ITG when comparing before and after chemotherapy. As mentioned above, the dFC variability changes after chemotherapy were consistent with the sFC changes.

Interestingly, we found some significant brain FC patterns that were related to chemotherapy only when using dFC analysis, while sFC analysis did not reveal any significant correlations. For example, increased dFC variability between the bilateral DLPFC and left precuneus was observed before chemotherapy, and decreased dFC between the right DLPFC and left precuneus was found after chemotherapy. The precuneus, which is part of the posteromedial parietal lobe, is involved in attentive tracking, visuospatial imagination, and spatially guided behaviors (Cavanna and Trimble 2006). Simo et al. (2018) revealed that lung cancer patients after chemotherapy showed decreased FC in the left cuneus and precuneus compared to HCs. Additionally, Wang et al. (2016) found increased sFC between the right DLPFC and the right precuneus after chemotherapy. We hypothesize that dFC analysis seems to capture more complex FC changes than sFC, which is also supported by the links that have been drawn between FC and the underlying brain structural connectivity.

In addition, after clustering analysis, we found that the dFC patterns were completely different under various connectivity states and increased dFC was even found in some states after chemotherapy. For example, in state 3, dFC was significantly increased between the left DLPFC and right MCG. However, we could not discover these FC pattern changes during sFC analysis. Fiorenzato et al. (Fiorenzato et al. 2019) also revealed two entirely different connectivity states in Parkinson’s disease patients. The cingulate cortex is usually perceived to be part of the limbic cortex, which lies immediately above the corpus callosum, and the MCG has been termed the cingulate motor area and can be activated by pain and errors made in many tasks (Kolling et al. 2016; Rolls 2019). Our results demonstrated that dFC analysis could capture new and latent changes in FC and that these changes were possibly derived from the dynamics of connectivity states. Even more importantly, these aforementioned findings provide evidence of increased MCG activity in lung cancer patients and allow us to speculate that this activity is a kind of compensatory mechanism in the brain activated when responding to cancer and chemotherapy.

Moreover, significant between-group differences in the temporal properties of dFC states were recognized in several states. Our results showed that lung cancer patients after chemotherapy tended to have a shortened MDT in certain states and a reduced NT between states, which have not been mentioned in previous studies. However, Fiorenzato et al. (Fiorenzato et al. 2019) found that an extended MDT in the segregated state and a reduced NT between states in Parkinson’s disease. Moreover, as we all know that the lung cancer patients are generally treated with platinum-based chemotherapy, which is related to increased cell death and decreased cell division in the central nervous system (Dietrich et al. 2006). Therefore, the destruction of neural structures in lung cancer patients receiving platinum-based chemotherapy is likely a reason for the decreases in the MDT and NT. Thus, we may reasonably conjecture that the MDT and NT could be reference indexes to assess the effects of chemotherapy on human-brain FC pattern. Hence, more comprehensive forward-looking studies are desperately needed to explore whether the MDT and NT can be appropriate potential biomarkers of cognitive impairment in lung cancer patients.

In this study, the MoCA scores of lung cancer patients after chemotherapy were dramatically reduced. Concerning the correlations between the FC and MoCA scores, we found no significant correlations between MoCA scores and sFC, while negative correlations between MoCA scores and altered dFC were observed in some brain regions. Static measures of FC, which provide a measure of brain function averaged over several minutes, are oversimplified, and dynamic FC measures that capture temporal changes in brain function on the order of a few seconds have been proposed. Therefore, we judged that dFC could capture more information and was more sensitive for assessing brain function and cognitive defects in lung cancer patients after chemotherapy than sFC.

There were a few limitations in this study. First, the relatively small sample size might reduce the generalizability of our results to some extent. Second, although the present study employed a longitudinal experimental design, follow-up data were collected only for patients in the lung cancer group and not for healthy controls. Previous studies (Feng et al. 2019; Pergolizzi et al. 2019) have provided evidence that cancer patients after chemotherapy exhibit FC changes during short-term follow-up (3–6 months) and have no significant difference in healthy controls. Nevertheless, all enrolled subjects should be strictly followed in subsequent longitudinal studies. Finally, we selected the bilateral DLPFC as the seed regions to investigate the sFC and dFC patterns in the ECN after chemotherapy and explore the correlational relationships with MoCA scores. The MoCA is a brief cognitive screening tool that is not very sensitive to certain domains, such as executive functions and memory, which have been exposed to be damaged after chemotherapy in previous studies. Thus, a more sophisticated and niche-targeted cognitive evaluation should be performed in future studies.

In conclusion, this is the first study to explore the sFC and dFC variations within the ECN and the relationships between the aberrant FC and cognitive decline in lung cancer patients before and after chemotherapy. Despite the limitations of the present study, our results revealed that using a dynamic connectivity analysis was more effective and sensitive for linking brain FC patterns and chemotherapy than methods that assume static brain states. The most important implication from our study is to bring awareness to cancer clinicians so they can provide information on expected CRCI to cancer patients. Moreover, several treatments are underway to help combat the symptoms of chemobrain. We concluded that the temporal dynamics of FC could be a potential biomarker to detect cognitive alterations in lung cancer patients receiving chemotherapy and provide a solid evidence for multidisciplinary rehabilitation prevention and treatment of chemobrain.
